# Outcomes of a Novel Surgery: Gastrojejunal–Ileal Interposition with Bipartition and Sleeve Gastrectomy for Type 2 Diabetes and Obesity

**DOI:** 10.3390/jcm15114027

**Published:** 2026-05-22

**Authors:** Tugrul Demirel, Necdet Sut, Surendra Ugale

**Affiliations:** 1 Department of General Surgery, Faculty of Medicine, Trakya University, Edirne 22030, Turkey; 2 Department of Biostatistics and Medical Informatics, Faculty of Medicine, Trakya University, Edirne 22030, Turkey; nsut@trakya.edu.tr; 3 Department of Advanced Laparoscopy & Bariatric-Metabolic Surgery, Kirloskar Hospital, Hyderabad 500004, India; surenugale@gmail.com

**Keywords:** gastrojejunal–ileal interposition, metabolic surgery, type 2 diabetes, bariatric surgery, diabetes remission

## Abstract

**Background/Objectives:** Gastrojejunal–ileal interposition with bipartition and sleeve gastrectomy (GJIB-SG) is a novel metabolic procedure developed to combine functional foregut exclusion with hindgut stimulation while preserving duodenal continuity and endoscopic biliary access. This study evaluated the medium-term glycemic, weight-loss, and nutritional safety outcomes of GJIB-SG in patients with obesity and long-standing type 2 diabetes (T2D). **Methods:** A retrospective single-center cohort of 30 consecutive patients with obesity and T2D who underwent GJIB-SG between January 2016 and August 2019 and reached at least 60 months of postoperative follow-up was analyzed at baseline and at 12, 24, 36, 48, and 60 months. Longitudinal data were analyzed by repeated-measures ANOVA with Greenhouse–Geisser correction and Bonferroni-adjusted pairwise comparisons. Diabetes remission was classified using the 2021 American Diabetes Association consensus definition (A1C < 6.5%, medication-free). **Results:** Mean body weight decreased from 102.4 ± 13.6 kg preoperatively to 73.5 ± 7.6 kg at 60 months (*p* < 0.001; mean %TWL 27.4%, mean %EWL 99.4%). Mean A1C decreased from 9.4 ± 1.6% to 6.0 ± 1.4% at 60 months (*p* < 0.001). Complete medication-free remission was achieved by 70.0% of patients at 12 months and 44.8% at 60 months; cumulatively, 25 of 30 (83.3%) achieved complete remission at one or more intervals, and 3 patients (10.0%) never achieved A1C < 6.5%. Triglycerides, total cholesterol, and LDL cholesterol decreased by 56%, 39%, and 35%, respectively. No protein–energy malnutrition or hypoalbuminemia occurred; however, a late rise in parathyroid hormone and a return of 25-OH vitamin D toward preoperative insufficient values by 60 months indicate the need for sustained micronutrient surveillance. One cardiovascular death at 24 months was not considered procedure related. **Conclusions:** In this single-center cohort, GJIB-SG was associated with durable weight loss, sustained glycemic improvement with cumulative complete remission in 83.3% of patients, and absence of severe nutritional complications over 60 months. Prospective comparative studies with longitudinal mixed-effects analysis are warranted to define the role of GJIB-SG within the metabolic–surgical armamentarium.

## 1. Introduction

Type 2 diabetes (T2D) represents one of the most formidable global health challenges of the 21st century, the prevalence of which is rising rapidly. Current estimates project a 45% global increase in diabetes over the next 25 years, largely driven by the prevalence of obesity [1].

Despite advances in pharmacotherapy and intensive lifestyle interventions, long-term glycemic control remains suboptimal for many patients. According to a recent statement from the American Diabetes Association, during 2015–2018, only 50.5% of U.S. community-dwelling adults with diabetes achieved the recommended glycated hemoglobin (A1C) target of <7%, while just 47.7% met the blood pressure goal of <130/80 mmHg, and 55.7% reached lipid control (defined as non-HDL cholesterol <130 mg/dL) [2]. Alarmingly, only 22.2% of adults achieved all three-risk factor targets concurrently. These data underscore the persistent difficulty of achieving comprehensive metabolic control under conventional medical therapy, despite the availability of modern pharmacologic and behavioral interventions. Conventional management is further limited by poor long-term adherence, progressive β-cell decline, and frequent weight regain, resulting in continued disease progression and elevated risk of both microvascular and macrovascular complications. Consequently, there is an urgent need for therapeutic strategies that address the underlying metabolic dysfunction and offer durable remission rather than transient glycemic improvement [2].

Over the past two decades, bariatric and metabolic surgery has fundamentally transformed the therapeutic paradigm for obesity-related T2D. Multiple studies have demonstrated that surgical interventions such as Roux-en-Y gastric bypass (RYGB), biliopancreatic diversion (BPD), and sleeve gastrectomy (SG) are superior to pharmacological/behavioral therapy alone in achieving sustained glycemic normalization, substantial weight reduction, and improved cardiovascular outcomes [3,4,5]. These effects are mediated not only by caloric restriction and weight loss but also by complex metabolic and hormonal mechanisms involving alterations in gut hormone secretion, bile acid metabolism, and nutrient sensing in RYGB and Duodenal Switch (DS). Furthermore, these procedures that combine restrictive and malabsorptive components—particularly those incorporating foregut exclusion and enhanced hindgut nutrient stimulation—have demonstrated the superior metabolic efficacy and durability of T2D remission compared with restrictive techniques like SG alone [5].

Gastrojejunal–ileal interposition with bipartition and sleeve gastrectomy (GJIB-SG) represents an innovative metabolic surgery technique designed to maximize the physiological advantages of SG, RYGB, BPD, transit bipartition (TB) and duodeno-jejunal–ileal interposition combining both the functional exclusion of foregut and stimulation of hindgut without eliminating food passage from the proximal intestines, while maintaining endoscopic access to duodenum. This hybrid configuration aims to optimize glycemic regulation and sustain weight loss by integrating gastric restriction with selective intestinal rerouting that enhances distal nutrient exposure and incretin-mediated insulin response. Importantly, one of the principal design features and therapeutic advantages of GJIB-SG is its safety profile in minimizing protein–energy malabsorption, distinguishing it from more malabsorptive procedures such as biliopancreatic diversion or distal bypass procedures, like SADI-S (single anastomosis duodeno-ileal bypass with sleeve) or SASI (single anastomosis sleeve-ileal bypass). By preserving adequate nutrient absorption while harnessing potent metabolic effects, GJIB-SG seeks to achieve durable T2D remission and weight loss without compromising nutritional integrity and endoscopic access to duodenum. The present study describes the surgical technique of GJIB-SG and evaluates its clinical and metabolic outcomes in comparison with established bariatric procedures, emphasizing T2D remission rates, the durability of metabolic improvement, safety, and weight loss efficacy relative to both conventional medical and surgical standards of care.

## 2. Materials and Methods

This study aimed to retrospectively evaluate the metabolic outcomes of patients who underwent gastrojejunal–ileal interposition with bipartition and sleeve gastrectomy (GJIB-SG) for obesity and type II diabetes (T2D) between January 2016 and August 2020, with annual follow-up thereafter. The diagnosis of type 2 diabetes (T2D) was established according to the criteria of the American Diabetes Association [2]. Between the same dates, the senior surgeon performed more than 1000 primary bariatric and metabolic procedures across three institutions, predominantly sleeve gastrectomy, together with transit bipartition, duodenoileal interposition with sleeve gastrectomy, Roux-en-Y and one-anastomosis gastric bypass, biliopancreatic diversion with duodenal switch, duodenojejunal bypass with sleeve gastrectomy, and GJIB-SG. Of these, 35 patients underwent GJIB-SG as a primary operation. The analytic cohort for the present analysis comprises 30 of these patients who had reached at least 60 months of follow-up; details are provided in Section 2.6. Patients who received GJIB-SG as a revisional procedure for protein–energy malnutrition after transit bipartition have been reported separately by our group [6] and were excluded here. Eligibility criteria were applied uniformly across all candidates, and the specific operation was selected through shared decision making after detailed counseling on the mechanisms, expected outcomes, and long-term implications of each available procedure. All patients provided written informed consent, including consent for the academic use of intraoperative recordings (captured without patient-identifying information). The institutional ethics committee approved the study.

### 2.1. Inclusion and Exclusion Criteria

Patients were eligible when they met all of the following: a confirmed diagnosis of T2D under pharmacological treatment for at least three years; A1C ≥ 7.0% despite ongoing antidiabetic therapy; preserved pancreatic β-cell reserve, defined as one-hour postprandial C-peptide > 2 ng/mL on a standardized mixed-meal tolerance test; age below 70 years; weight stability (body-weight variation ≤ 3% during the preceding three months); and written informed consent acknowledging the investigational nature of the procedure. The cohort was predominantly composed of patients with obesity (BMI ≥ 30 kg/m^2^); however, three overweight patients (BMI 25.0–29.9 kg/m^2^) were enrolled on an individualized basis after multidisciplinary review, on the grounds of favorable β-cell reserve and a low diabetes severity and remission score (DSS), both of which predict greater metabolic benefit. A single patient with A1C 6.9% was likewise accepted owing to morbid obesity and persistent dependence on antidiabetic pharmacotherapy.

The exclusion criteria were latent autoimmune or type 1 diabetes (excluded clinically on the basis of low postprandial C-peptide); prior bariatric, metabolic, or major upper gastrointestinal surgery; pregnancy or planned conception; active malignancy or other life-limiting illness; advanced cardiopulmonary disease precluding general anesthesia; severe renal impairment; ongoing psychiatric instability or untreated eating disorders; current substance-use disorder; and obesity attributable to a secondary endocrine cause.

### 2.2. Study Outcome Measures

The primary outcomes were weight loss and metabolic outcomes, assessed at predefined postoperative follow-up intervals up to 60 months in the analytic cohort (Section 2.6). Weight-loss–related parameters were calculated at each follow-up interval according to standard definitions, as detailed in Appendix A.

Complete remission of type 2 diabetes (T2D) was defined per the 2021 American Diabetes Association consensus statement [2], as A1C < 6.5% maintained for ≥3 months in the absence of glucose-lowering medication.

The secondary outcomes encompassed longitudinal metabolic and nutritional parameters (fasting and postprandial plasma glucose, A1C, hematocrit, hemoglobin, blood urea nitrogen, creatinine, iron, folic acid, vitamin B12, vitamin D3, parathyroid hormone, albumin, triglycerides, total cholesterol, LDL cholesterol, HDL cholesterol), end-organ involvement, and medication requirements.

### 2.3. Patient Selection, Evaluation, and Preoperative Preparation

All patients underwent a comprehensive preoperative evaluation, including detailed medical history, physical examination, and laboratory assessment (biochemistry, fasting blood glucose, lipid profile, thyroid function, cortisol).

Pancreatic β-cell functional reserve was assessed using a mixed-meal tolerance test, with measurement of fasting and 1 h postprandial C-peptide, insulin, and blood glucose levels following a standardized balanced meal. Patients demonstrating postprandial C-peptide levels > 2 ng/mL were deemed eligible for inclusion.

End-organ complications were recorded as binary variables (present or absent) at baseline and each follow-up interval. Retinopathy was identified by documented diabetic retinopathy on prior ophthalmologic examination, with longitudinal status updated by continued specialist follow-up. Nephropathy was identified by referring-nephrologist diagnosis of diabetic kidney disease, with longitudinal status updated using available serum creatinine values and clinical history. Peripheral neuropathy and erectile dysfunction were recorded by patient self-report, without standardized validated instruments (e.g., Michigan Neuropathy Screening Instrument, International Index of Erectile Function), specialist examination, or electrodiagnostic testing. Obstructive sleep apnea was identified by documented continuous positive airway pressure (CPAP) use, with longitudinal status updated by reported continuation or discontinuation; repeat polysomnography was not performed. No central adjudication, standardized re-evaluation protocol, or formal pre-versus-post statistical testing was applied.

Upper gastrointestinal endoscopy was routinely performed in all patients. Abdominal ultrasonography was used to evaluate hepatic steatosis and to exclude gallstones. No preoperative bowel preparation or low-calorie diet aimed at liver volume reduction was implemented. Pharmacological prophylaxis against deep vein thrombosis was initiated 12 h before surgery using subcutaneous low-molecular-weight heparin.

A previously validated DSS [7] was used to assess preoperative disease severity, based on age, BMI, diabetes duration, presence of microvascular and macrovascular complications, insulin requirement, and stimulated C-peptide. The cumulative score was calculated for each patient prior to surgery.

### 2.4. Surgical Technique

**Figure 1:**
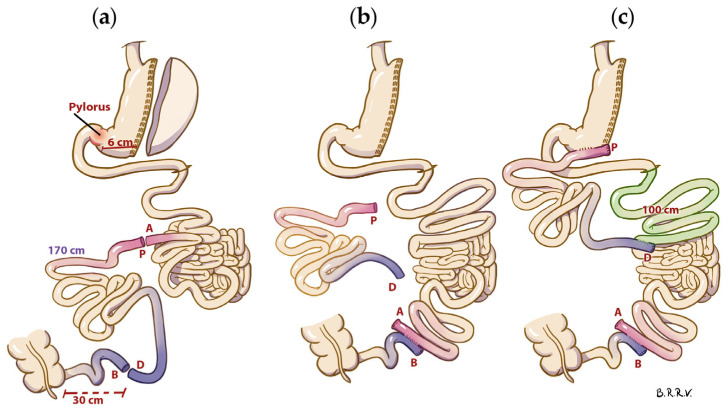
Steps of gastrojejunal–ileal interposition with bipartition and sleeve gastrectomy (GJIB-SG). (**a**) After completion of sleeve gastrectomy, an ileal segment is isolated by transecting the ileum at 30 cm and 200 cm proximal to the ileocecal valve. (**b**) Distal intestinal continuity is re-established through a side-to-side anastomosis between the distal end of the proximal bowel [A] and the proximal tip of the ileal loop in connection with the ileocecal valve [B]. (**c**) The ileal interposition is completed by anastomosing the proximal tip of the isolated ileal segment [P] to the gastric antrum, and the distal tip [D] to the proximal jejunum, located 100 cm distal to the ligament of Treitz. Anatomical labels: A, distal end of the proximal small bowel; B, proximal tip of the ileal loop adjacent to the ileocecal valve; P, proximal tip of the isolated ileal interposition segment; D, distal tip of the isolated ileal interposition segment.

All procedures were performed by the same surgeon. The procedure begins with a sleeve gastrectomy. An ileal segment of approximately 170 cm is isolated by two consecutive transections of the ileum at 30 cm and 200 cm from the ileocecal valve with vascular cartridges (Figure 1) as defined by De Paula [8,9].

Intestinal continuity of the distal bowel was restored by a side-to-side ileoileostomy between the proximal small bowel (from the Treitz side) and the distal ileum at 30 cm from the ileocecal valve. The proximal end of the interposed ileal segment was then brought in an antecolic fashion to the gastric sleeve, and a side-to-side stapled gastroileostomy with an anastomotic length of approximately 4–5 cm was created to the posterior gastric wall. The final anastomosis was performed between the distal end of the ileal segment (double-legged suture) and the previously marked jejunal site 100 cm distal to the ligament of Treitz. Transections and anastomoses were performed using 60 mm vascular (2.5 mm) cartridges from one of two systems: the Echelon Flex stapler (Ethicon, Johnson & Johnson, Cincinnati, OH, USA) or the Endo GIA stapler (Medtronic, Minneapolis, MN, USA), according to availability across the participating institutions.; the stapler orifices of the anastomoses were closed with continuous 3/0 polydioxanone sutures (PDS II, Ethicon, Johnson & Johnson, Cincinnati, OH, USA). All mesenteric defects were meticulously closed using 3/0 polypropylene sutures (Prolene, Ethicon, Johnson & Johnson, Cincinnati, OH, USA). The procedure was based on the ileal-segment preparation described by De Paula [9,10]. The completed reconstruction is depicted in Figure 1.

A complete intraoperative video of the GJIB-SG procedure, with stepwise on-screen annotations, is openly available via the Figshare repository [10]. 

### 2.5. Postoperative Supplementation and Follow-Up

All patients received a standardized postoperative supplementation regimen. During the first postoperative year, this comprised an oral multivitamin twice daily, an effervescent calcium–vitamin D combination tablet (1000 mg of calcium carbonate/880 IU of cholecalciferol) once daily for the first six months and twice weekly thereafter, oral ferrous sulfate at 80 mg daily, and an additional 5000 IU of cholecalciferol daily. After the first postoperative year, continued supplementation was recommended on an individualized basis for up to five years, with adjustments based on annual laboratory monitoring. Adherence to long-term supplementation declined after the first postoperative year; re-prescription was undertaken selectively in patients with biochemical evidence of micronutrient or vitamin D insufficiency.

Clinical follow-up was scheduled every three months during the first postoperative year, every six months during the second year, and annually thereafter. Annual laboratory monitoring included calcium, alkaline phosphatase, parathyroid hormone, and 25-OH vitamin D in addition to the metabolic and nutritional parameters reported below. The majority of follow-up encounters were conducted face-to-face; patients residing internationally were maintained in active follow-up through structured video consultations supplemented by laboratory results submitted from external referring institutions. Outcomes were assessed at each visit by the senior surgeon (T.D.) and the attending clinical team; no blinded outcome assessor or central adjudication committee was used. Some peripheral laboratory results were not consistently retrievable, contributing to variability in laboratory completeness across analytes; per-variable analytic and reporting conventions are detailed in the corresponding table footnotes in the Results section.

### 2.6. Statistical Analysis

All statistical analyses were conducted using IBM SPSS Statistics (version 20.0, IBM Corp., Armonk, NY, USA). Numerical variables are presented as mean ± standard deviation (SD), and categorical variables are expressed as number (percentage). Where additional descriptive context was clinically informative, the minimum–maximum range is reported alongside the mean ± SD; the specific variables for which range information is presented are indicated in the relevant table.

The analytic cohort comprised 30 consecutively enrolled patients who underwent GJIB-SG between January 2016 and August 2019 and who had reached at least 60 months of postoperative follow-up at the time of analysis. All 30 patients attended every scheduled follow-up visit; however, laboratory measurement completeness varied by analyte and timepoint. Missing values represent observations that were genuinely not obtained and were not imputed. Per-variable analytic samples and reporting conventions are detailed in the footnotes in the Results section.

Longitudinal continuous variables were analyzed using the General Linear Models (GLM) repeated-measures ANOVA framework, with timepoint as the within-subjects factor (six levels: baseline and 12, 24, 36, 48, and 60 months). Listwise deletion was applied within each variable; the analytic sample size for each parameter is therefore the number of patients with a complete six-timepoint record for that analyte. Sphericity was assessed by Mauchly’s test for each variable; when sphericity was violated, the Greenhouse–Geisser correction was applied. When the omnibus repeated-measures effect was statistically significant, follow-up means were compared with the baseline measurement (reference category) using within-subjects simple contrasts. Pairwise comparisons among all six timepoints were additionally examined using estimated marginal means with Bonferroni adjustment, and the resulting adjusted *p*-values are reported as appropriate in the relevant Results tables and in the corresponding text.

Bonferroni adjustment was applied to pairwise comparisons among timepoints within each outcome; no correction was applied across the family of outcome variables, and primary conclusions are anchored on the principal metabolic outcomes—glycemic control, weight loss, and medication burden—where the magnitudes of change are large and consistent across follow-up intervals.

Diabetes remission was classified at each annual visit according to the 2021 American Diabetes Association consensus statement [2], using concurrent A1C values and active antidiabetic medication status. Three mutually exclusive categories were defined: complete remission (A1C < 6.5% maintained for at least three months in the absence of glucose-lowering medication), A1C < 6.5% on reduced antidiabetic therapy (A1C below the diabetes threshold but with continued use of insulin and/or oral antidiabetic agents), and no remission (A1C ≥ 6.5%). Remission durability was characterized descriptively as cross-sectional point-prevalence at each annual interval, cumulatively (the proportion of patients ever achieving remission), and conditionally (the proportion of ever-remitters in remission at each subsequent visit). Formal time-to-relapse analysis using survival methods was not performed, given the small number of relapse events; relapse patterns are reported descriptively in Section 3.3. Remission classifications reflect status at scheduled annual visits; brief intercurrent medication use between visits would not be captured by this data structure.

All tests were two-tailed, and a *p*-value < 0.05 was considered statistically significant.

## 3. Results

### 3.1. Patient Cohort and Operative Outcomes

The demographic, anthropometric, metabolic, and operative characteristics of the analytic cohort are summarized in Table 1. Two patients (6.7%) were in the overweight range (BMI 25.0–29.9) and were enrolled on an individualized basis after multidisciplinary review, on the basis of favorable β-cell reserve and a low diabetes severity score (DSS). A single patient with a baseline A1C of 6.9% was likewise accepted owing to morbid obesity and persistent dependence on antidiabetic pharmacotherapy. Pancreatic β-cell reserve, evaluated by mixed-meal tolerance testing, was preserved in all 30 patients: all met the inclusion threshold of postprandial C-peptide > 2 ng/mL.

The operation was completed laparoscopically in all patients. There were no intraoperative complications, no conversions to open surgery, and no major perioperative complications (Clavien–Dindo grade ≥ III) within 30 days of the index operation. Five patients (16.7%) underwent concomitant intraoperative cholecystectomy at the time of GJIB-SG for previously documented cholelithiasis; no other concomitant procedures were performed.

During the 60-month follow-up period, one patient (3.3%) developed persistent postoperative dumping syndrome, which resolved spontaneously within two years without surgical or pharmacologic intervention. No other patient developed chronic diarrhea or severe gastrointestinal intolerance. One patient (3.3%) developed adhesive small bowel obstruction at 20 months postoperatively, attributable to a fibrotic adhesive band that produced an ischemic jejunal segment; this required open exploratory laparotomy, segmental jejunal resection, and reanastomosis, with preservation of the transposed ileal segment. The same patient subsequently underwent open mesh repair of an incisional hernia at the laparotomy site approximately one year later, the hernia having developed at the wound of the open adhesiolysis rather than at the original laparoscopic port sites. One patient (3.3%) died of acute myocardial infarction approximately 24 months postoperatively; the death was attributed to underlying cardiovascular disease present preoperatively and was not considered to be a consequence of the surgical procedure or its metabolic effects. Cumulative all-cause mortality during the 60-month analytic window was 1/30 (3.3%), and one patient (3.3%) underwent surgical intervention for a late complication of the index operation.

### 3.2. Weight Loss Outcomes

Weight loss after GJIB-SG was rapid in the first postoperative year and was sustained throughout the 60-month follow-up period. The mean body weight decreased from 102.4 ± 13.6 kg preoperatively to 71.4 ± 8.1 kg at 12 months—the period of maximal weight loss—and remained essentially stable thereafter, with mean weights of 71.5 ± 7.5 kg at 24 months, 72.0 ± 7.6 kg at 36 months, 73.2 ± 7.7 kg at 48 months, and 73.5 ± 7.6 kg at 60 months. The reduction from baseline was statistically significant at every follow-up interval (*p* < 0.001 at all timepoints; Bonferroni-adjusted pairwise comparisons of estimated marginal means from repeated-measures ANOVA with Greenhouse–Geisser correction). The mean body mass index followed the same pattern, declining from 35.5 ± 5.0 kg/m^2^ (range 26.5–47.1) at baseline to 24.7 ± 2.2 kg/m^2^ (range 21.3–28.4) at 12 months and 25.4 ± 2.1 kg/m^2^ (range 22.7–29.7) at 60 months. The 60-month mean BMI placed the cohort in the upper end of the normal-weight category, and the individual patient BMIs at 60 months ranged from normal-weight to upper-overweight.

The mean total weight loss was 31.0 ± 13.1 kg at 12 months and 28.9 ± 12.5 kg at 60 months, corresponding to a mean percentage of total weight loss of 29.5 ± 9.8% at 12 months and 27.4 ± 9.4% at 60 months. The mean percentage of excess weight lost was 108.4 ± 28.7% at 12 months—exceeding 100% because several patients lost more than their preoperative excess weight relative to ideal body weight—and 99.4 ± 32.7% at 60 months. The trajectory of these parameters across follow-up indicates that the small post-nadir weight regain observed between 12 and 60 months (a mean of 2.1 kg, or approximately 2 percentage points of %TWL) was modest in magnitude relative to the dominant early reduction, and that the cohort retained nearly all of its initial weight loss at the 60-month endpoint. Detailed values for all anthropometric and weight-loss parameters at each follow-up interval are presented in Table 2.

### 3.3. Glycemic Control and Diabetes Remission

**Figure 2:**
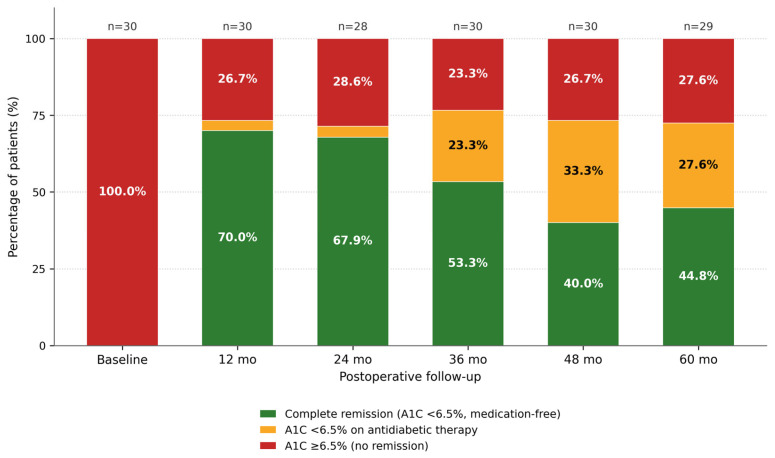
Longitudinal distribution of glycemic response status following GJIB-SG. At each follow-up visit, patients with available A1C data were classified into one of three mutually exclusive categories per the 2021 American Diabetes Association consensus definitions: complete remission (A1C < 6.5% maintained for at least three months without glucose-lowering medication), A1C < 6.5% on reduced antidiabetic therapy (A1C below the diabetes threshold but with continued use of insulin and/or oral antidiabetic agents), and no remission (A1C ≥ 6.5%). Bars represent the percentage distribution of these categories at each timepoint. Denominators (*n*) above each column refer to the number of patients with A1C measurements available at the corresponding visit; two patients at 24 months and one at 60 months had missing A1C values and are not included in those columns’ denominators. The 12-month and 24-month “A1C < 6.5% on reduced therapy” segments represent a single patient each (3.3% and 3.6%, respectively) and are not labeled in the figure for visual clarity. The rising prevalence of the intermediate category from 36 months onward reflects the post-nadir reintroduction of antidiabetic therapy in patients whose A1C remained below the diabetes threshold.

Glycemic control improved markedly and was sustained throughout the 60-month follow-up period. The mean A1C decreased from 9.4 ± 1.6% preoperatively to 6.1 ± 1.0% at 12 months and remained stable at every subsequent interval, reaching 6.0 ± 1.4% at 60 months (*p* < 0.001 vs. baseline at all timepoints; Bonferroni-adjusted pairwise comparisons of estimated marginal means from repeated-measures ANOVA with Greenhouse–Geisser correction). The mean fasting plasma glucose decreased from 233.4 ± 75.5 mg/dL at baseline to 102.7 ± 30.4 mg/dL at 12 months and 103.4 ± 38.8 mg/dL at 60 months (*p* < 0.001 at all timepoints). The mean postprandial glucose decreased from 326.9 ± 73.4 mg/dL preoperatively to 136.7 ± 51.7 mg/dL at 12 months and 133.4 ± 60.5 mg/dL at 60 months (*p* < 0.001 at all timepoints). Detailed glycemic and remission outcomes at each follow-up interval are presented in Table 3 and visualized in Figure 2.

Diabetes remission status was classified at each follow-up visit using the 2021 American Diabetes Association consensus definitions (Section 2.6). The point-prevalence complete remission—A1C < 6.5% maintained without glucose-lowering medication—was 21/30 (70.0%) at 12 months, 19/28 (67.9%) at 24 months, 16/30 (53.3%) at 36 months, 12/30 (40.0%) at 48 months, and 13/29 (44.8%) at 60 months. The decline in point-prevalence remission between 12 and 48 months was accompanied by a corresponding rise in the proportion of patients with A1C < 6.5% on reduced antidiabetic therapy, which increased from 1/30 (3.3%) at 12 months to 10/30 (33.3%) at 48 months and 8/29 (27.6%) at 60 months. The proportion of patients with A1C ≥ 6.5% (no remission) remained relatively stable across follow-up, ranging from 23.3% to 28.6% of patients with available A1C at each visit. This pattern indicates that the post-nadir declines in strict medication-free remission predominantly reflected the reintroduction of antidiabetic therapy in patients whose A1C remained below the diabetes threshold, rather than progression to overt hyperglycemia.

Cumulatively, 25 of 30 patients (83.3%) achieved complete medication-free remission at one or more follow-up intervals, and 27 of 30 patients (90.0%) achieved A1C < 6.5% at one or more intervals irrespective of medication status. Among the 25 patients who ever achieved complete remission, 13 (52.0%) remained in complete remission at the 60-month visit, indicating that the durability of strict medication-free remission was incomplete in approximately half of ever-remitters over five years. The mean A1C remained well below the baseline value across the cohort at every follow-up timepoint regardless of remission status, reflecting substantial residual glycemic benefit even in patients who did not maintain complete medication-free remission.

Among the eight patients with A1C ≥ 6.5% at the 60-month visit, three never achieved A1C < 6.5% at any follow-up interval, and five reached A1C < 6.5% at one or more interim timepoints before subsequently exceeding the diabetes threshold. This pattern indicates that incomplete durability of glycemic remission may be at least as important to long-term outcome in this cohort as failure of initial induction. The three never-remitters shared a profile of moderately advanced disease, with preoperative DSSs of 10–11, baseline A1C values of 8.8–10.8%, and a ten-year diabetes duration; one of the three had a borderline preoperative postprandial C-peptide reserve at 2.37 ng/mL, while the other two demonstrated preserved β-cell reserve. The five patients with late escape from remission showed heterogeneous baseline profiles and trajectories, with A1C excursions occurring between 36 and 60 months. These observations are descriptive and were not formally tested in a multivariable predictive model owing to sample-size constraints; the limited cohort precludes inference about whether disease severity, β-cell reserve, or other baseline factors predict either failure of induction or failure of durability.

### 3.4. Nutritional Parameters

Throughout the 60-month follow-up period, no patient reached a body mass index below 21 kg/m^2^ and none met the World Health Organization criterion for underweight (<18.5 kg/m^2^). The lowest individual BMI observed during follow-up was 21.3 kg/m^2^, recorded in one patient at the 12-month assessment. No case of protein–energy malnutrition was documented. The mean serum albumin showed a small, transient decrease during early follow-up, declining from 4.45 ± 0.43 g/dL preoperatively to 4.07 ± 0.47 g/dL at 12 months (*p* < 0.05 vs. baseline) and returning to baseline thereafter (4.49 ± 0.38 g/dL at 60 months); all the individual albumin values at every interval remained above 3.5 g/dL, and no clinically relevant hypoalbuminemia was observed at any timepoint. The mean hemoglobin and mean serum creatinine showed no clinically meaningful change across follow-up; detailed values are presented in Table 4.

Longitudinal micronutrient values are summarized in Table 4. The mean serum iron remained within the laboratory reference range across follow-up (74.8 ± 39.2 µg/dL at baseline, 86.5 ± 48.1 µg/dL at 60 months), without a statistically significant change from baseline at any timepoint after Bonferroni adjustment. The mean serum vitamin B12 fluctuated mildly across follow-up (429 ± 259 pg/mL at baseline, 496 ± 506 pg/mL at 60 months) without a significant change from baseline, and no patient developed symptomatic B12 deficiency. The mean serum folate increased from 7.7 ± 4.4 ng/mL at baseline to 11.0 ± 3.5 ng/mL at 60 months, consistent with effective oral folic-acid supplementation, although the change did not reach statistical significance after Bonferroni correction in the listwise analytic sample (*n* = 13). The mean 25-OH vitamin D3 rose from 19.8 ± 13.4 ng/mL preoperatively to 34.3 ± 23.1 ng/mL at 12 months under intensive postoperative supplementation and then declined gradually to 18.3 ± 5.9 ng/mL at 60 months, returning to a value comparable to the preoperative measurement. The mean parathyroid hormone (PTH) rose from 35.8 ± 12.1 pg/mL at baseline to 55.0 ± 41.9 pg/mL at 24 months and 50.4 ± 17.2 pg/mL at 60 months, with statistical significance versus baseline reached at the 48-month interval (*p* < 0.05); the wide standard deviation at the 24-month timepoint reflects a single patient with a transient peak value of 212 pg/mL that returned to the laboratory reference range at subsequent visits, and the median PTH at 24 months was 42.2 pg/mL. Although the mean PTH values remained largely within the laboratory reference range, the longitudinal trajectory between 24 and 60 months together with the parallel late decline in 25-OH vitamin D is consistent with early or subclinical secondary hyperparathyroidism. The timing of the PTH rise corresponds to the period during which the standardized supplementation protocol is tapered (Section 2.5). No patient developed frank biochemical or clinical secondary hyperparathyroidism requiring targeted pharmacological intervention during follow-up.

Improvements in lipid parameters were substantial and sustained throughout follow-up. The mean triglyceride levels decreased from 222.9 ± 145.0 mg/dL preoperatively to 102.0 ± 29.3 mg/dL at 12 months and remained at 96.8 ± 19.8 mg/dL at 60 months—a 56% reduction from baseline (*p* < 0.05 vs. baseline at all follow-up timepoints after Bonferroni adjustment). The mean total cholesterol decreased from 203.9 ± 40.8 mg/dL to 125.2 ± 35.9 mg/dL at 60 months (39% reduction; *p* < 0.001 at all timepoints), and the mean LDL cholesterol decreased from 126.5 ± 33.0 mg/dL to 81.8 ± 20.1 mg/dL at 60 months (35% reduction; *p* < 0.001 at most timepoints, *p* < 0.05 at 36 months). The mean HDL cholesterol rose modestly from 36.9 ± 8.3 mg/dL at baseline to 42.7 ± 8.4 mg/dL at 60 months (*p* < 0.05 at the 60-month visit). Detailed lipid trajectories are presented in Table 4.

No severe protein–energy malnutrition or hypoalbuminemia was observed in this cohort. However, incomplete and variable micronutrient surveillance—particularly for folate (analytic *n* = 13) and 25-OH vitamin D (analytic *n* = 19)—together with the longitudinal PTH trajectory and the persistent low mean vitamin D values at 60 months limits firm conclusions about long-term nutritional safety.

### 3.5. Comorbidities and Cardiovascular Risk Profile

Reductions in the pharmacologic treatment of metabolic comorbidities were observed across follow-up. Among the 23 patients (76.7%) receiving antihypertensive therapy at baseline, 22 had discontinued by 12 months and the entire cohort was off antihypertensive medication from 24 months onward. Among the 18 patients (60.0%) receiving antilipidemic therapy at baseline, all had discontinued by 12 months, and none required reinitiation during the 60-month follow-up period. Of the 12 patients (40.0%) using continuous positive airway pressure (CPAP) for obstructive sleep apnea preoperatively, all reported discontinuation of CPAP at the 12-month visit and at every subsequent annual interval. As described in Table 5, the baseline classifications for these conditions reflect the diagnosed condition, whereas the follow-up classifications reflect the status of pharmacologic or device-based treatment; resolution is therefore reported as the absence of ongoing treatment requirement rather than as independent re-adjudication of the underlying condition.

Diabetes-related end-organ complications also declined across follow-up. Diabetic neuropathy was reported in 15 patients (50.0%) preoperatively and in 1 to 2 patients (3.3–6.7%) at each follow-up interval. Diabetic retinopathy was present in 8 patients (26.7%) at baseline and in 1 patient at 12 months, with no patient classified as actively retinopathic at 24 to 60 months. Diabetic nephropathy was present in 1 patient at baseline; the same patient remained classified as nephropathic at every subsequent follow-up visit, and no new case of nephropathy emerged during the 60-month observation period. Among the 21 male patients, erectile dysfunction was reported by 16 (76.2%) preoperatively and by 7 (33.3%) at each follow-up interval from 12 to 60 months. As described in Section 2.3 and Table 5, retinopathy and nephropathy were ascertained from clinical history and available laboratory parameters, and neuropathy and erectile dysfunction were ascertained primarily from patient self-reports; reported improvements should therefore be interpreted as descriptive longitudinal observations rather than as objectively re-adjudicated clinical outcomes.

Cardiovascular surrogate markers showed substantial improvement. From baseline to 60 months, the mean A1C decreased by 3.4 percentage points, mean LDL cholesterol by 47 mg/dL, mean triglycerides by 126 mg/dL, and mean body weight by 29 kg, while mean HDL cholesterol increased by 6 mg/dL. Direct cardiovascular endpoints—myocardial infarction, stroke, cardiovascular death, and heart failure—were not systematically ascertained in the present cohort, and one patient died of acute myocardial infarction at approximately 24 months postoperatively (Section 3.1). The observed surrogate-marker improvements describe a favorable cardiovascular risk factor profile but do not establish a reduction in hard cardiovascular outcomes. Comorbidity status, end-organ classifications, and changes in pharmacologic treatment are summarized in Table 5.

## 4. Discussion

This retrospective analysis of 30 individuals with obesity and type 2 diabetes undergoing sleeve gastrectomy combined with gastrojejunal–ileal interposition with bipartition (GJIB-SG) describes a sustained improvement in glycemic control and diabetes-related comorbidities over a 60-month follow-up period. The procedure was associated with a marked reduction in the use of insulin and oral antidiabetic agents, with durable weight loss and no observed protein–energy malnutrition or chronic diarrhea. These findings are descriptive of a single-center cohort and are not derived from a comparative design.

The metabolic surgical literature provides important context for interpreting these results, although direct comparison with our cohort is precluded by the absence of a concurrent comparator arm. Among established procedures, Roux-en-Y gastric bypass (RYGB) has been reported to produce greater diabetes remission and weight loss than sleeve gastrectomy alone in randomized comparisons [11], while biliopancreatic diversion (BPD) and one-anastomosis gastric bypass (OAGB) have been associated with the highest remission rates and weight-loss durability across the spectrum, often at the cost of greater nutritional impact [3,12,13,14,15,16,17,18,19,20]. GJIB-SG was conceived to combine functional foregut exclusion and hindgut stimulation while preserving anatomical access to the duodenum and avoiding the malabsorptive load of fully diversionary procedures. Whether this design intent translates into favorable comparative outcomes can only be tested in future prospective comparative studies.

Among published RYGB cohorts, Queiroz et al. reported in a comprehensive meta-analysis that RYGB was associated with approximately three times greater odds of diabetes remission than sleeve gastrectomy alone, with consistently greater weight loss across randomized trials [11]. In the five-year Oseberg randomized controlled trial [21], complete diabetes remission was achieved in 50% and partial remission in 63% of RYGB patients, with mean total body weight loss of 22.2% and improved lipid parameters; postprandial hypoglycemia occurred in 28% of patients [21]. A contemporary single-center cohort of 34 RYGB patients reported a one-year decline in mean A1C from 9.7% to 5.7%, a 25.6% total weight loss, and 82.4% complete remission, accompanied by a 26% incidence of anemia [22].

Biliopancreatic diversion (BPD) represents the most metabolically potent end of the spectrum: Scopinaro et al. reported diabetes remission rates of 98–100% at ten years and excess weight loss exceeding 70% in long-term follow-up [20]. In Mingrone et al.’s ten-year randomized trial comparing RYGB and BPD with medical therapy, complete diabetes remission was achieved in 50% of RYGB and BPD patients combined versus 5.5% with pharmacological/behavioral treatment alone, with mean A1C remaining well controlled (≤6.7%) despite partial relapse in more than half of initially remitted cases [19]. BPD’s metabolic potency is, however, accompanied by substantial nutritional impact: protein–energy malnutrition was reported in up to 30% of early cases, later reduced to ~11% after procedural modifications [20]. Among OAGB cohorts, Kermansaravi et al. reported significantly greater 1-year and 5-year excess weight loss and a 28% higher 5-year remission rate compared with both RYGB and SG, suggesting enhanced long-term metabolic durability [17].

In the present cohort, complete diabetes remission per the 2021 ADA consensus criteria (A1C < 6.5% in the absence of glucose-lowering medication) was achieved in 70.0% of patients at one year and 44.8% at five years. On a cumulative basis, 25 of 30 patients (83.3%) achieved complete medication-free remission at one or more follow-up intervals, and 27 of 30 patients (90.0%) achieved A1C < 6.5% at one or more intervals regardless of medication status, identifying a clinically meaningful subset of delayed responders. Conditional remission persistence among ever-remitters was 84.0% at 12 months, declining to 48.0% at 48 months and 54.2% at 60 months; the post-nadir declines reflected medication reintroduction in patients whose A1C remained below the diabetes threshold rather than progression to overt hyperglycemia. Our cumulative complete remission of 83.3% lies in the upper range of published reports across metabolic procedures, while our point-prevalence at 60 months of 44.8% sits below the corresponding RYGB and BPD figures, possibly reflecting the partial reintroduction of antidiabetic therapy in patients whose A1C remained below the diabetes threshold rather than failure of metabolic effect; cohort-level differences in patient selection, baseline severity, remission definitions, follow-up schedules, and outcome assessment preclude direct comparative inference. Lipid normalization in our cohort was substantial, with reductions of approximately 56%, 39%, and 35% in mean triglycerides, total cholesterol, and LDL cholesterol from baseline to 60 months; serum albumin remained within the normal range at every interval, and no patient developed protein–energy malnutrition.

In total, 8 of 30 patients (26.7%) had A1C ≥ 6.5% at the 60-month visit. Three of these patients never achieved A1C < 6.5% at any follow-up interval; the remaining five reached A1C < 6.5% at one or more interim timepoints before subsequently exceeding the diabetes threshold. The three never-remitters shared a profile of moderately advanced disease, with preoperative DSSs of 10–11, baseline A1C values of 8.8–10.8%, and ten-year diabetes duration; one of the three had borderline preoperative postprandial C-peptide reserve at 2.37 ng/mL, while the other two demonstrated preserved β-cell reserve. The five patients with late escape from remission showed heterogeneous baseline profiles and A1C excursions occurring between 36 and 60 months. These observations are descriptive and were not formally tested in a multivariable predictive model owing to sample-size constraints; the limited cohort precludes inference about whether disease severity, β-cell reserve, or other baseline factors predict either failure of induction or failure of durability, and whether patients with very advanced disease would be better served by alternative metabolic procedures, such as duodenoileal interposition, requires direct comparative testing in prospective studies.

GJIB-SG shares anatomical features with transit bipartition (TB) [17,23,24,25,26,27,28,29,30,31] and duodenoileal interposition with diverted sleeve gastrectomy (DII-SG) [8,9,32,33,34,35,36], and its differences from these procedures are relevant to its design rationale. Transit bipartition, introduced by Santoro [31], has been described in numerous cohort studies, particularly in its single-anastomosis variants (SASI) [37,38,39,40,41,42,43,44] and loop bipartition [25,45,46]. One feature shared by GJIB-SG and TB is preservation of the pyloroduodenal complex, which permits future endoscopic access to the biliary tree—a clinically valuable property absent from procedures involving duodenal division such as duodenal switch, duodenoileal interposition, BPD, and RYGB. GJIB-SG additionally implements the functional limitation of nutrient flux through the proximal duodenum, reproducing the foregut exclusion mechanism [32,35,47,48] without sacrificing endoscopic access. Severe protein–energy malabsorption with refractory diarrhea has been reported after TB and has occasionally required revisional surgery [39,44,49,50,51], and in one cohort, 6.8% of patients required conversion to sleeve gastrectomy due to uncontrolled excessive weight loss [38]. We have separately reported the use of GJIB-SG as a salvage procedure in patients with protein–energy malnutrition and intractable diarrhea following TB, with normalization of nutritional parameters and preservation of metabolic efficacy [6]. In the present primary-surgery cohort, no patient developed protein–energy malnutrition or hypoalbuminemia, and no patient required revisional surgery for nutritional or weight-related complications.

Long-term follow-up data on transit bipartition remain limited, with most published series reporting follow-up of less than two years [23,24,27,29,30,37,39,40,41,42,43,50,52], a few extending beyond three years [28,38], and the longest series five years [31]. The present study contributes a 60-month complete follow-up dataset, with all 30 analyzed patients evaluated at every annual interval; this addresses a gap in the existing TB/DII-SG literature, in which late-follow-up sample sizes are often substantially smaller than baseline.

The longitudinal trajectory of parathyroid hormone observed in our cohort—rising from 35.8 ± 12.1 pg/mL at baseline to mean values of approximately 47–55 pg/mL between 24 and 60 months, without frank biochemical or clinical secondary hyperparathyroidism—illustrates a recurring challenge in long-term post-bariatric care: the divergence between protocolized supplementation and real-world adherence beyond the first postoperative year. The parallel pattern in 25-OH vitamin D—an early postoperative rise to 34.3 ± 23.1 ng/mL on intensive supplementation followed by a gradual return toward baseline insufficient values by 60 months (18.3 ± 5.9 ng/mL)—supports the same interpretation. Sustained patient education, structured re-prescription at annual visits, and active surveillance of bone-mineral status—including calcium, alkaline phosphatase, PTH, and 25-OH vitamin D, ideally complemented by periodic bone densitometry—should be considered integral components of long-term care after GJIB-SG. The absence of systematic DEXA bone-density assessment in the present retrospective cohort is a limitation we plan to address in prospective evaluations of the procedure.

### Limitations

*Methodological design and statistical limitations*: This is a retrospective single-center cohort without a concurrent comparator arm, which precludes causal or comparative interpretation. The cohort of 30 patients limits inferential power for rare events, multivariable adjustment, and predictive modeling of remission failure or relapse. Repeated-measures ANOVA was performed on the balanced 60-month cohort; mixed-effects modeling, more appropriate for unbalanced longitudinal data, is planned for future prospective evaluations of this procedure. Per-variable analytic samples varied with laboratory completeness, and missing values were not imputed; inferential conclusions for parameters with smaller analytic samples (folate *n* = 13, 25-OH vitamin D *n* = 19) should be interpreted with appropriate caution.

*Outcome ascertainment and scope*: End-organ complications, including retinopathy, nephropathy, neuropathy, erectile dysfunction, and obstructive sleep apnea, were ascertained from clinical history, available laboratory parameters, and patient self-reports rather than from standardized validated instruments or central adjudication. Hard cardiovascular endpoints were not systematically assessed, and one cardiovascular death occurred at approximately 24 months postoperatively (Section 3.1); the surrogate-marker improvements observed in this cohort do not establish a reduction in hard cardiovascular outcomes. DEXA bone-density assessment, structured dietary intake, and body-composition measurement were not performed. Latent autoimmune diabetes was excluded clinically and by C-peptide criteria rather than by formal anti-GAD testing. The findings should be interpreted as preliminary observational evidence supporting prospective comparative evaluation rather than as evidence of comparative efficacy or safety.

## 5. Conclusions

In this single-center retrospective cohort of 30 patients with obesity and long-standing type 2 diabetes, gastrojejunal–ileal interposition with bipartition and sleeve gastrectomy (GJIB-SG) was associated with durable weight loss, sustained improvement in glycemic control with cumulative complete remission in 83.3% of patients, and an absence of severe protein–energy malnutrition or hypoalbuminemia over a 60-month follow-up period. The procedure preserves duodenal continuity, allowing for future endoscopic biliary access, and combines functional foregut exclusion with early hindgut stimulation. These observational findings support the feasibility and short- to medium-term safety of GJIB-SG as a metabolic surgical option in carefully selected patients. The absence of a concurrent comparator arm precludes causal or comparative interpretation; prospective comparative studies with standardized follow-up, formal confounder adjustment, and longitudinal mixed-effects statistical methods will be necessary before strong conclusions regarding superiority, broad clinical adoption, or definitive efficacy can be drawn.

## Figures and Tables

**Table 1:** Baseline demographic, anthropometric, metabolic, and operative characteristics of the analytic cohort (*n* = 30).

Characteristic	Value
Patient Demographics	
Age, mean ± SD (range), years	52.5 ± 10.3 (34–69)
Sex, male/female, *n*	21/9
Diabetes duration, mean ± SD (range), years	9.2 ± 3.3 (3–15)
diabetes severity score (DSS), mean ± SD (range)	9.5 ± 1.5 (7–13)
Anthropometric	
Weight, mean ± SD, kg	102.4 ± 13.6
Body mass index, mean ± SD (range), kg/m^2^	35.5 ± 5.0 (26.5–47.0)
BMI category, *n* (%)	
Overweight (BMI 25.0–29.9)	2 (6.7%)
Class I obesity (BMI 30.0–34.9)	14 (46.7%)
Class II obesity (BMI 35.0–39.9)	8 (26.7%)
Class III obesity (BMI ≥ 40)	6 (20.0%)
Baseline Glycemic Parameters	
A1C, mean ± SD (range), %	9.4 ± 1.6 (6.9–12.7)
Fasting plasma glucose, mean ± SD, mg/dL	231.6 ± 73.3
Postprandial plasma glucose, mean ± SD, mg/dL	331.2 ± 72.3
Pancreatic β-Cell Reserve	
Fasting C-peptide, mean ± SD (range), ng/mL	3.30 ± 1.33 (1.45–6.75)
Postprandial C-peptide, mean ± SD (range), ng/mL	5.35 ± 1.88 (2.37–9.32)
Fasting insulin, mean ± SD, µIU/mL	20.2 ± 11.4
Postprandial insulin, mean ± SD, µIU/mL	47.0 ± 25.7
Baseline Antidiabetic Therapy, *n* (%)	
Insulin therapy	20 (66.7%)
Oral antidiabetic therapy	27 (90.0%)
Baseline Comorbidities and End-Organ Status, *n* (%)	
Hypertension	23 (76.7%)
Dyslipidemia	18 (60.0%)
Obstructive sleep apnea	12 (40.0%)
Diabetic neuropathy	15 (50.0%)
Diabetic retinopathy	8 (26.7%)
Diabetic nephropathy	1 (3.3%)
Erectile dysfunction (males)	16/21 (76.2%)
Operative Characteristics	
Operative time, mean ± SD (range), minutes	172.2 ± 44.1 (117–289)
Postoperative hospital stay, mean ± SD (range), days	4.6 ± 0.7 (3–6)
Concomitant intraoperative cholecystectomy, *n* (%)	5 (16.7%)

All values are presented for the analytic cohort of 30 patients defined in Section 2.6. Continuous variables are shown as mean ± standard deviation, with range (minimum–maximum) for selected variables; categorical variables are shown as number (percentage). The diabetes severity score (DSS) is a composite preoperative score reflecting glycemic control, diabetes duration, and pharmacologic burden, as defined in Section 2.3. Fasting and postprandial C-peptide and insulin were measured preoperatively for β-cell function characterization and were not repeated during follow-up. Baseline insulin measurements were available for 26 patients (fasting) and 24 patients (postprandial); all other baseline parameters were available for the entire cohort except where indicated. Erectile dysfunction was assessed only among the 21 male patients of the cohort. Abbreviations: SD, standard deviation; BMI, body mass index; A1C, glycated hemoglobin.

**Table 2:** Longitudinal changes in anthropometric and weight-loss parameters following GJIB-SG.

Parameter	Baseline	12 mo	24 mo	36 mo	48 mo	60 mo
*n*	30	30	30	30	30	30
Weight, mean ± SD, kg	102.4 ± 13.6	71.4 ± 8.1 †	71.5 ± 7.5 †	72.0 ± 7.6 †	73.2 ± 7.7 †	73.5 ± 7.6 †
BMI, mean ± SD (range), kg/m^2^	35.5 ± 5.0 (26.5–47.1)	24.7 ± 2.2 (21.3–28.4) †	24.7 ± 2.0 (21.9–29.0) †	24.9 ± 2.1 (21.9–29.7) †	25.3 ± 2.1 (22.7–29.7) †	25.4 ± 2.1 (22.7–29.7) †
TWL, mean ± SD, kg	—	31.0 ± 13.1	30.9 ± 13.1	30.4 ± 13.2	29.2 ± 12.6	28.9 ± 12.5
%TWL, mean ± SD	—	29.5 ± 9.8	29.3 ± 9.8	28.9 ± 9.9	27.7 ± 9.5	27.4 ± 9.4
%EWL, mean ± SD	—	108.4 ± 28.7	106.8 ± 26.9	105.2 ± 26.2	100.6 ± 32.6	99.4 ± 32.7

This table presents the longitudinal evolution of anthropometric and weight-loss parameters at scheduled postoperative follow-up intervals after GJIB-SG. All 30 patients had complete weight and BMI data at every follow-up interval. Continuous variables are shown as mean ± standard deviation. † *p* < 0.001 versus baseline (Bonferroni-adjusted pairwise comparisons of estimated marginal means from repeated-measures ANOVA). TWL, %TWL, and %EWL are calculated as change from preoperative baseline and are therefore reported descriptively from 12 months onward without inferential comparison to baseline. An em-dash (—) indicates that the parameter is not applicable at baseline. Abbreviations: mo, months; TWL, total weight loss (kg); %TWL, percentage of total weight loss; %EWL, percentage of excess weight loss; BMI, body mass index.

**Table 3:** Longitudinal glycemic outcomes and diabetes remission following GJIB-SG.

Parameter	Baseline	12 mo	24 mo	36 mo	48 mo	60 mo
Glycemic Parameters						
A1C mean ± SD (range), %	9.4 ± 1.6 (6.9–12.7)	6.1 ± 1.0 (4.7–8.6) †	6.0 ± 1.0 (4.5–8.3) †	5.9 ± 1.3 (4.2–10.5) †	6.1 ± 1.4 (4.2–10.3) †	6.0 ± 1.4 (4.1–10.3) †
Fasting glucose, mean ± SD, mg/dL	233.4 ± 75.5	102.7 ± 30.4 †	104.1 ± 26.3 †	103.4 ± 34.7 †	110.4 ± 34.1 †	103.4 ± 38.8 †
Postprandial glucose, mean ± SD, mg/dL	326.9 ± 73.4	136.7 ± 51.7 †	128.6 ± 56.0 †	130.7 ± 60.5 †	140.4 ± 61.2 †	133.4 ± 60.5 †
Diabetes Remission Classification, *n*/*N* (%)						
Complete remission (A1C < 6.5%, medication-free) *	0/30 (0%)	21/30 (70.0%)	19/28 (67.9%)	16/30 (53.3%)	12/30 (40.0%)	13/29 (44.8%)
A1C < 6.5% on reduced antidiabetic therapy *	0/30 (0%)	1/30 (3.3%)	1/28 (3.6%)	7/30 (23.3%)	10/30 (33.3%)	8/29 (27.6%)
A1C ≥ 6.5% (no remission)	30/30 (100%)	8/30 (26.7%)	8/28 (28.6%)	7/30 (23.3%)	8/30 (26.7%)	8/29 (27.6%)

Glycemic parameters were analyzed by repeated-measures ANOVA on the cohort of 30 patients, with listwise deletion within each variable; analytic samples were *n* = 27 for A1C, *n* = 28 for fasting glucose, and *n* = 26 for postprandial glucose. Missing values were genuinely missing observations and were not imputed. Sphericity was assessed by Mauchly’s test, and the Greenhouse–Geisser correction was applied when sphericity was violated. Pairwise comparisons among timepoints used estimated marginal means with Bonferroni adjustment. † *p* < 0.001 versus baseline. Diabetes remission classifications were derived patient-by-patient from concurrent A1C values and active antidiabetic medication status (insulin and/or oral antidiabetic agent); denominators (*N*) at each interval refer to patients with A1C available at that visit. * Diabetes remission rates are reported under two complementary definitions in accordance with the 2021 American Diabetes Association consensus statement [7]. “Complete remission” is defined as A1C < 6.5% maintained for at least three months in the absence of glucose-lowering medication. “A1C < 6.5% on reduced antidiabetic therapy” is reported separately as a descriptor of significant glycemic improvement that does not meet the strict ADA medication-free criterion. Patients with A1C ≥ 6.5% at the corresponding visit are classified as not in remission. Abbreviations: mo, months; A1C, glycated hemoglobin.

**Table 4:** Longitudinal metabolic, nutritional, and safety laboratory parameters following GJIB-SG.

Parameter	Baseline	12 mo	24 mo	36 mo	48 mo	60 mo
Hematologic and Renal						
Hemoglobin, mean ± SD, g/dL	14.1 ± 1.6	12.3 ± 2.3 †	12.1 ± 2.1 †	12.7 ± 2.3	12.9 ± 2.0 ‡	12.8 ± 1.6 ‡
Creatinine, mean ± SD, mg/dL	0.89 ± 0.28	0.85 ± 0.27	0.93 ± 0.29	0.95 ± 0.34	0.93 ± 0.35	0.95 ± 0.28
Lipid Panel						
Triglycerides, mean ± SD (range), mg/dL	222.9 ± 145.0 (108–597)	102.0 ± 29.3 (54–189) ‡	102.7 ± 29.1 (56–175) ‡	102.2 ± 33.1 (50–168) ‡	95.1 ± 27.2 (56–151) ‡	96.8 ± 19.8 (41–162) ‡
Total cholesterol, mean ± SD (range), mg/dL	203.9 ± 40.8 (137–324)	128.0 ± 30.1 (77–174) †	132.8 ± 36.0 (91–247) †	135.1 ± 42.3 (89–263) †	126.8 ± 42.2 (72–236) †	125.2 ± 35.9 (67–189) †
LDL cholesterol, mean ± SD, mg/dL	126.5 ± 33.0	81.8 ± 15.5 †	83.0 ± 25.1 †	76.6 ± 26.1 ‡	71.3 ± 20.3 †	81.8 ± 20.1 †
HDL cholesterol, mean ± SD, mg/dL	36.9 ± 8.3	39.8 ± 6.5	42.4 ± 8.8	41.1 ± 7.4	41.8 ± 7.9	42.7 ± 8.4 ‡
Nutritional and Safety						
Albumin, mean ± SD, g/dL	4.45 ± 0.43	4.07 ± 0.47 ‡	4.22 ± 0.45	4.25 ± 0.43	4.34 ± 0.38	4.47 ± 0.38
Iron, mean ± SD, µg/dL §	74.8 ± 39.2	68.9 ± 33.1	64.9 ± 29.1	59.5 ± 23.1	69.4 ± 29.4	86.5 ± 48.1
Folic acid, mean ± SD, ng/mL	7.7 ± 4.4	10.6 ± 4.3	8.3 ± 4.4	8.0 ± 3.4	9.8 ± 4.3	11.0 ± 3.5
Vitamin B12, mean ± SD, pg/mL	429 ± 259	606 ± 394	383 ± 224	445 ± 496	446 ± 307	496 ± 506
Vitamin D3 (25-OH), mean ± SD, ng/mL	19.8 ± 13.4	34.3 ± 23.1	28.8 ± 23.4	22.8 ± 14.2	24.1 ± 10.4	18.3 ± 5.9
PTH, mean ± SD, pg/mL	35.8 ± 12.1	40.6 ± 16.9	55.0 ± 41.9	51.0 ± 20.0	52.9 ± 20.3 ‡	50.4 ± 17.2

Longitudinal values are reported as mean ± SD on listwise samples used for repeated-measures ANOVA: hemoglobin, creatinine, triglycerides, HDL, vitamin B12 (*n* = 22–23); albumin, LDL, PTH (*n* = 21); total cholesterol (*n* = 25); vitamin D3 (*n* = 19); folic acid (*n* = 13). § Iron is reported as the mean ± SD of all available observations at each timepoint (per-timepoint *n* = 20, 27, 26, 28, 27, 29) because the listwise sample (*n* = 15) was not representative of the cohort; the inferential statistics for iron are nevertheless computed on the listwise sample, which showed no significant change from baseline at any interval. Ranges given for triglycerides and total cholesterol are computed on all available observations and reflect the full cohort distribution. All 30 patients reached the 60-month follow-up visit; analytic samples differ from 30 because laboratory completeness varied by analyte. Missing values were genuinely missing observations and were not imputed. Sphericity was assessed by Mauchly’s test, and the Greenhouse–Geisser correction was applied when sphericity was violated. Pairwise comparisons among timepoints used estimated marginal means with Bonferroni adjustment. † *p* < 0.001 versus baseline; ‡ *p* < 0.05 versus baseline. Cells without an asterisk did not reach significance versus baseline. Abbreviations: mo, months; LDL, low-density lipoprotein cholesterol; HDL, high-density lipoprotein cholesterol; PTH, parathyroid hormone.

**Table 5:** Longitudinal follow-up of antidiabetic medication use, end-organ complications, and comorbid conditions following GJIB-SG.

Parameter	Baseline	12 mo	24 mo	36 mo	48 mo	60 mo
*n*	30	30	30	30	30	30
Antidiabetic medication use, *n* (%)						
Insulin therapy	20 (66.7%)	2 (6.7%)	1 (3.3%)	2 (6.7%)	5 (16.7%)	6 (20.0%)
Oral antidiabetic therapy	27 (90.0%)	1 (3.3%)	1 (3.3%)	6 (20.0%)	10 (33.3%)	11 (36.7%)
Comorbid conditions, *n* (%) ^1^						
Hypertension ^2^	23 (76.7%)	1 (3.3%)	0 (0%)	0 (0%)	0 (0%)	0 (0%)
Dyslipidemia ^2^	18 (60.0%)	0 (0%)	0 (0%)	0 (0%)	0 (0%)	0 (0%)
Obstructive sleep apnea ^3^	12 (40.0%)	0 (0%)	0 (0%)	0 (0%)	0 (0%)	0 (0%)
End-organ complications, *n* (%) ^4^						
Diabetic neuropathy	15 (50.0%)	1 (3.3%)	1 (3.3%)	1 (3.3%)	1 (3.3%)	2 (6.7%)
Diabetic nephropathy	1 (3.3%)	1 (3.3%)	1 (3.3%)	1 (3.3%)	1 (3.3%)	1 (3.3%)
Diabetic retinopathy	8 (26.7%)	1 (3.3%)	0 (0%)	0 (0%)	0 (0%)	0 (0%)
Erectile dysfunction (males) ^5^	16/21 (76.2%)	7/21 (33.3%)	7/21 (33.3%)	7/21 (33.3%)	7/21 (33.3%)	7/21 (33.3%)

All 30 patients had complete medication and clinical-status data at every interval. Values reported as number (percentage); denominators (*n*) refer to the analytic cohort of 30 patients except where indicated. Data are descriptive; no statistical comparisons were performed. ^1^ At baseline, comorbid conditions are recorded as the presence of the diagnosed condition. At follow-up intervals, presence is recorded by ongoing pharmacologic treatment for the condition, or, for obstructive sleep apnea, by continued CPAP use. Resolution at follow-up therefore reflects the discontinuation of pharmacological therapy or CPAP rather than independent clinical re-adjudication of the underlying condition. ^2^ Hypertension and dyslipidemia at follow-up reflect the proportion of patients on antihypertensive or antilipidemic pharmacotherapy at the corresponding visit. ^3^ Obstructive sleep apneas at follow-up reflects the proportion of patients with continued CPAP use at the corresponding visit; repeat polysomnography was not performed. ^4^ End-organ complications were ascertained as described in Section 2.3: retinopathy from documented diabetic retinopathy on prior ophthalmologic examination updated by continued specialist follow-up; nephropathy from referring-nephrologist diagnosis updated by available creatinine values; neuropathy and erectile dysfunction from patient self-reports without standardized validated instruments. No central adjudication or formal pre-versus-post statistical testing was applied. ^5^ Erectile dysfunction is reported among the 21 male patients of the cohort. Abbreviations: mo, months; CPAP, continuous positive airway pressure.

## Data Availability

The raw data supporting the conclusions of this article will be made available by the authors on request.

## Abbreviations

The following abbreviations are used in this manuscript:

T2D	type 2 diabetes
A1C	glycated hemoglobin
DSS	diabetes severity and remission score
LDL	low-density lipoprotein
HDL	high-density lipoprotein
SD	standard deviation
GJIB-SG	gastrojejunal–ileal interposition with bipartition and sleeve gastrectomy
RYGB	roux-en-y gastric bypass
OAGB	one-anastomosis gastric bypass
BPD	biliopancreatic diversion
DII-SG	duodenoileal interposition with diverted sleeve gastrectomy
SASI	single anastomosis sleeve ileal bypass
SG	sleeve gastrectomy
DS	duodenal switch
TB	transit bipartition
BMI	body mass index
EWL	excess weight loss
%EWL	percentage excess weight loss
%EBMIL	percentage excess BMI loss
%TWL	percentage total weight loss
PEM	protein–energy malnutrition
ERCP	endoscopic retrograde cholangiopancreatoscopy

## Appendix A. Definitions of Weight-Loss–Related Parameters

Weight-loss–related parameters were calculated at each follow-up interval according to the following standard definitions. Preoperative weight refers to the body weight measured at the time of surgical admission. Body mass index (BMI) was calculated as weight in kilograms divided by height in meters squared. Ideal body weight was anchored at the body weight corresponding to a BMI of 25 kg/m^2^ for the patient’s measured height. Excess weight was defined as the difference between preoperative weight and ideal body weight at BMI 25 kg/m^2^.

TWL (kg) = preoperative weight − weight at follow-up

%TWL = [TWL ÷ preoperative weight] × 100

Excess weight (kg) = preoperative weight − ideal body weight at BMI 25 kg/m^2^

%EWL = [TWL ÷ excess weight] × 100

Under this definition, %EWL values may exceed 100% in patients whose postoperative weight falls below the BMI-25 anchor; this pattern was observed in several patients in the present cohort and is reflected in the cohort-level mean %EWL values reported in Table 2.

*Abbreviations:* TWL, total weight loss; %TWL, percentage of total weight loss; %EWL, percentage of excess weight lost; BMI, body mass index.
